# Pneumococcal population dynamics: Investigating vaccine-induced changes through multiscale modelling

**DOI:** 10.1371/journal.pcbi.1011755

**Published:** 2023-12-28

**Authors:** Nicola Mulberry, Alexander R. Rutherford, Caroline Colijn

**Affiliations:** Department of Mathematics, Simon Fraser University, Burnaby, British Columbia, Canada; University of Zurich, SWITZERLAND

## Abstract

The mechanisms behind vaccine-induced strain replacement in the pneumococcus remain poorly understood. There is emerging evidence that distinct pneumococcal lineages can co-colonise for significant time periods, and that novel recombinants can readily emerge during natural colonisation. Despite this, patterns of post-vaccine replacement are indicative of competition between specific lineages. Here, we develop a multiscale transmission model to investigate explicitly how within host dynamics shape observed ecological patterns, both pre- and post-vaccination. Our model framework explores competition between and within strains defined by distinct antigenic, metabolic and resistance profiles. We allow for strains to freely co-colonise and recombine within hosts, and consider how each of these types may contribute to a strain’s overall fitness. Our results suggest that antigenic and resistance profiles are key drivers of post-vaccine success.

## Author summary

*Streptococcus pneumoniae* is a pathogen of major public health concern globally. Pneumococcal strains exhibit diversity in their capsular serotype, metabolic profiles, and properties of antibiotic resistance (among other traits). Pneumococcal conjugate vaccines have been successful at targeting invasive serotypes, thereby reducing disease. In the presence of vaccination programs, pneumococcal populations have been shown to undergo significant shifts, and we can use observed patterns of strain replacement at the population level to help understand competition at the within-host scale during (co-)colonisation. Using a nested model with explicit within-host dynamics, we show how competition between and within distinct types, type-specific immunity, and heterogeneity in duration of carriage may help explain patterns of vaccine-induced replacement.

## 1 Introduction

*Streptococcus pneumoniae* (the pneumococcus) is a leading cause of pneumonia, meningitis and sepsis globally, and hence a significant human pathogen. The first pneumococcal conjugate vaccines (PCV) were introduced in the year 2000. These vaccines target a small subset of the circulating serotypes: initially, 7 out of over 90. While these vaccines have been successful at reducing pneumococcal disease, the effect on overall carriage has been minimal [1, 2], indicative of strong competition among the circulating strains.

Observations of vaccine-induced changes can lead to key insights into the forces that shape pneumococcal population structure. Initial research showed compelling evidence for competition among serotypes, owing to the fact that certain non-vaccine types appear to benefit disproportionately to the elimination of vaccine-targeted serotypes [3]. A second indicator for pneumococcal competition comes from the emergence of so-called capsular switch variants post-vaccination [4]. The pneumococcus has the ability to “switch” its capsular serotype through genetic recombination, in some cases allowing a lineage which was previously associated to a vaccine-targeted serotype to persist post-vaccination [5]. Capsular switching events are thought to be commonplace, and while the origin of these variants often predate vaccine introduction [6], it is only after widespread vaccination that they are able to expand. It has been observed that capsular switch variants tend to coincide with those serotypes which expand most significantly post-vaccination [7].

There is evidence to suggest that co-colonisation by distinct pneumococcal lineages is commonplace [8], and that direct competition within hosts may not always be strong enough to exclude subsequent colonisation. Further insights into within-host dynamics are given by Chaguza *et al*. [9], who observe high strain diversity within hosts driven, in part, by homologous recombination. Understanding the within-host mechanisms that lead to observed patterns of strain structure and replacement at the host-population scale remains an open and interesting question.

Co-colonisation has been identified as a key mechanism driving stable coexistence in models of multistrain pathogens [10–13]. Co-colonisation drives coexistence by reducing competition among strains, and allowing strains with a higher within-host fitness to overtake a less-fit strain during the course of infection. Basic models that take into account within-host takeover (“superinfection” models [14]) generate coexistence, but rely on a strong separation between the within-host selection and transmission processes. For bacteria such as the pneumococcus, where co-colonisation has been observed on the order of weeks [9], such separation of time-scales is unrealistic.

Motivated by the pneumococcus in particular, multistrain compartmental models with co-colonisation classes and incremental take-over have been developed [12]. These models include that of Davies *et al*. [11], wherein drug-resistant and drug-sensitive pathogen strains are assumed to be in direct competition within hosts. The model of Lehtinen *et al*. [15] also investigates ecological patterns in the pneumococcus, focusing on how serotype-specific duration of carriage may be linked to the evolution of drug-resistance, but does not consider within-host competition. Alternatively, the model of Watkins *et al*. [5] was developed to investigate capsular switching events. In this model, the authors assume that strains that share key attributes (namely, an antigenic type and a metabolic type) are in direct competition and cannot co-colonise a host; in contrast, strains that share neither of these attributes are able to co-colonise and have no effect on the fitness of the other. Here, we seek to combine many ideas from these modelling approaches to develop a framework for investigating how within-host competition maintains key ecological patterns in the pneumococcus.

We develop a mechanistic mathematical model that describes the dynamics of interacting pneumococcal types explicitly at the within-host level during single and co-colonisation events. This nested model has strong, reciprocal feedback between scales, which allows us to examine explicitly how within-host dynamics shape population-level trends. We allow for recombination to occur during co-colonisation events to investigate whether or not population structure emerges over long time-scales, and to investigate under which conditions capsular switch variants are most likely to arise post-vaccination.

## 2 Results

**Fig 1:**
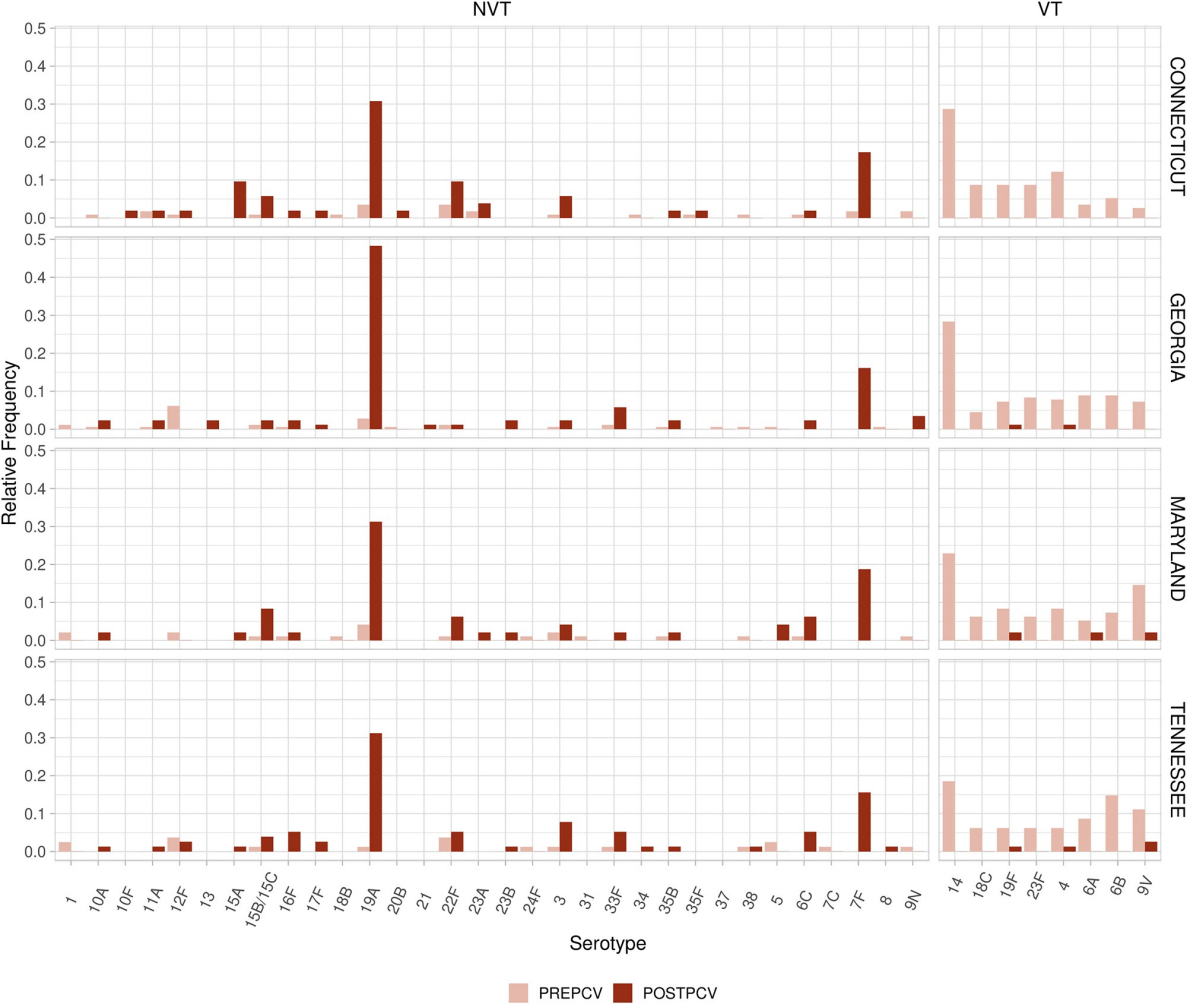
Changes in relative serotype frequencies due to vaccination. Each serotype is divided into vaccine types (VT) or non-vaccine types (NVT). The pre-PCV7 period is composed of three sample years from 1998–2000. The post-PCV7 period is from one sample period in 2009. These two sample periods had a roughly equal number of samples per region. We include 6A as a vaccine type due to the effectiveness of PCV7 at targeting this serotype [17].

We first investigate pre- and post-vaccination trends using pneumococcal sequences collected from across the United States as part of the “Active Bacterial Core surveillance” program. These data are available for download from https://data.monocle.sanger.ac.uk/. While this is only a small subset of the data available as part of the Global Pneumococcal Sequencing Project [16], these data are unique in that they span up to 9 years post-PCV7 introduction and show significant decrease in prevalence across all vaccine types (Fig 1).

**Fig 2:**
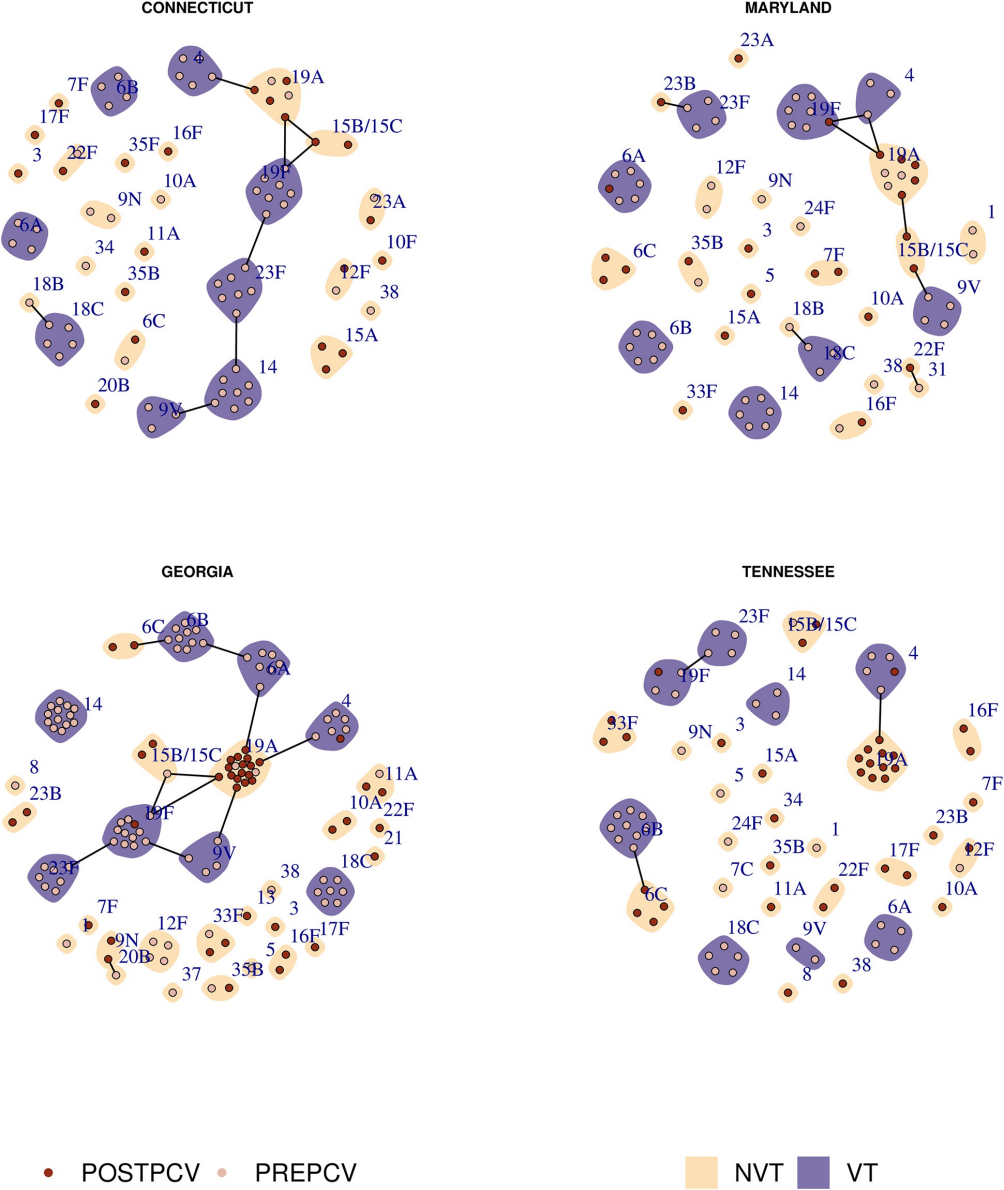
Pre- and post-PCV7 population structure in four US states. Each node represents a sequence type and each community (shaded patch) represents a serotype. Nodes are coloured according to the era of maximum sampled relative frequency, either pre- or post-PCV7. Communities are coloured according to whether or not the given serotype was a vaccine type (VT) or nonvaccine type (NVT). Edges represent a shared sequence type. Note that we include 6A as a vaccine type due to the effectiveness of PCV7 at targeting this serotype [17].

Previous research has hypothesized that exclusion occurs between sequence types (as defined through multi-locus sequence typing) due to direct metabolic competition [5, 18], and that the elimination of vaccine type (VT) competitors may allow a nonvaccine type (NVT) with the same metabolic profile to emerge post-vaccination. The sequence type of an isolate is defined by the allelic profile of specific core genes involved with metabolism. This typing method is thus better-suited to illustrating metabolic competition in the population rather than using lineages defined through alternative clustering methods (such as Global Pneumococcal Sequence Clusters [16]). In Fig 2, we show putative competition networks among the circulating serotypes in four US states. Each node represents a sampled sequence type within the indicated serotype, coloured by its dominant sample year (either pre- or post-PCV7), and grouped within its serotype. Edges denote a shared sequence type, and communities represent a shared serotype. During a single sample year, we observe that most sequence types are associated with a given serotype, and that the majority of serotypes are associated with only a few sequence types. However, in each region we see a multiple occurrences of putative capsular switching events between vaccine and nonvaccine types.

Our conceptual framework allows us to explicitly model metabolic competition on the within-host scale, and to investigate the combined effect of competition and duration of carriage at the host-population scale. We define strains by an antigenic type (AT), metabolic type (MT) and resistance type: for simplicity, either drug-resistant (R) or drug-sensitive (S). If two strains of the same MT co-colonise the same host, they compete directly against each other for resources. We model an AT-specific host immune response (determined by the AT-specific parameter *α*) which lasts on the order of 1–2 years per host. We assume that each MT is associated with an intrinsic within-host growth rate (*κ*), which may be furthermore affected by the strain’s resistance profile. We model a uniform population-level treatment process whereby any host may be put on treatment upon colonisation. The antigenic type, resistance type, in-host competition and host infection history all contribute to heterogeneity in duration of carriage and transmissibility. Antigenic types are furthermore classified into vaccine types (VTs) and nonvaccine types (NVTs). We model vaccination as coming in the form of a significant growth cost to the vaccine types, which enforces the gradual elimination of these strains at the host population level. Additional model details are given in Section 4.

**Fig 3:**
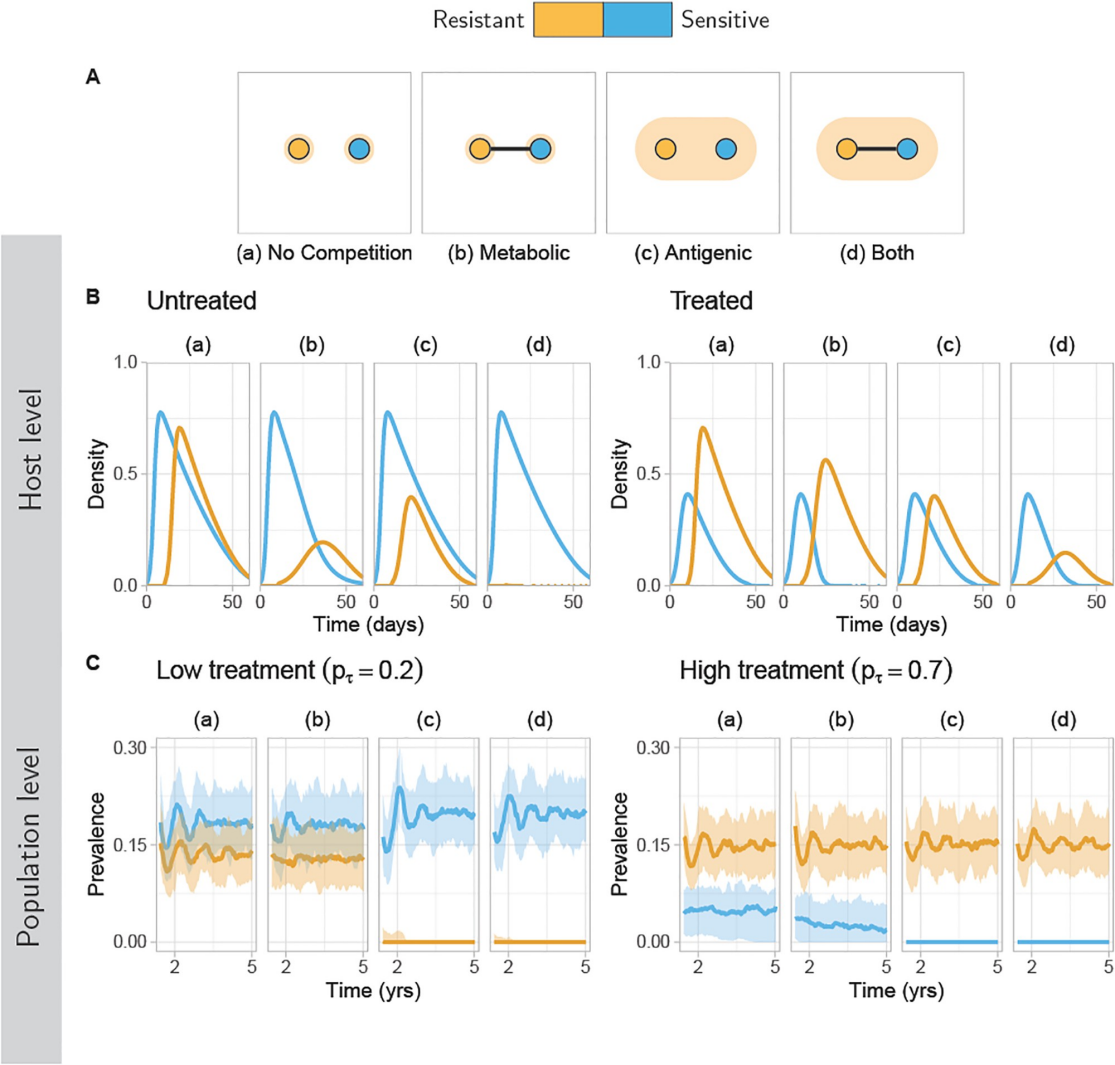
Co-colonisation of resistance and sensitivity at the within- and between-host scales. The dynamics of co-colonisation between drug-resistant and drug-sensitive strains are shown for the four different competition types. (A) Illustration of the system for each of the four competition types (a)–(d). (a) No competition between the two strains; (b) The two strains share a metabolic type; (c) The two strains share a serotype; (d) The two strains share both a metabolic type and an antigenic type. (B) Trajectories show the within-host prevalence of each strain, assuming that the resistant strain invades 10 days following an initial infection with the sensitive strain. Trajectories for each competition type under treatment (left) and under no treatment (right) are shown. (C) Trajectories at the population-level, taking one strain sampled from each host. Simulations use 2,000 hosts and are repeated 72 times. Lines show the median frequency of each strain over all simulations, bands show 95% quantiles. Trajectories for each competition type are shown under low treatment coverage (left) and high treatment coverage (right). Parameters are fixed with *c* = 0.15, *τ* = 0.35 and *β* = 0.1. For each antigenic type, we set *α* = 0.03.

We illustrate the short-term model dynamics in Fig 3 for two strains, one drug-sensitive and one drug-resistant, under the four different competition types. Here, the within-host model is simulated under the assumption that the sensitive strain is the primary coloniser, followed by the resistant strain after 10 days. The population level dynamics are simulated by linking the within-host model to a stochastic transmission model in a population of individuals (hosts). We observe how treatment and competition shape the within-host strain trajectories, and how, in turn, the within-host dynamics shape each strain’s population-level prevalence. If the two strains do not compete directly with each other, through either metabolic or antigenic competition, then neither one strain excludes the other in a co-colonisation event, and both strains can coexist at the population level. However, as we increase the competition between the two strains, we see cases where one strain may start to exclude the other, with the dominant resistance-type being determined as expected by the treatment coverage. Exclusion is particularly stark in the case of antigenic competition, whereas metabolic competition *on its own* appears to have a weaker effect on the population-level dynamics.

**Fig 4:**
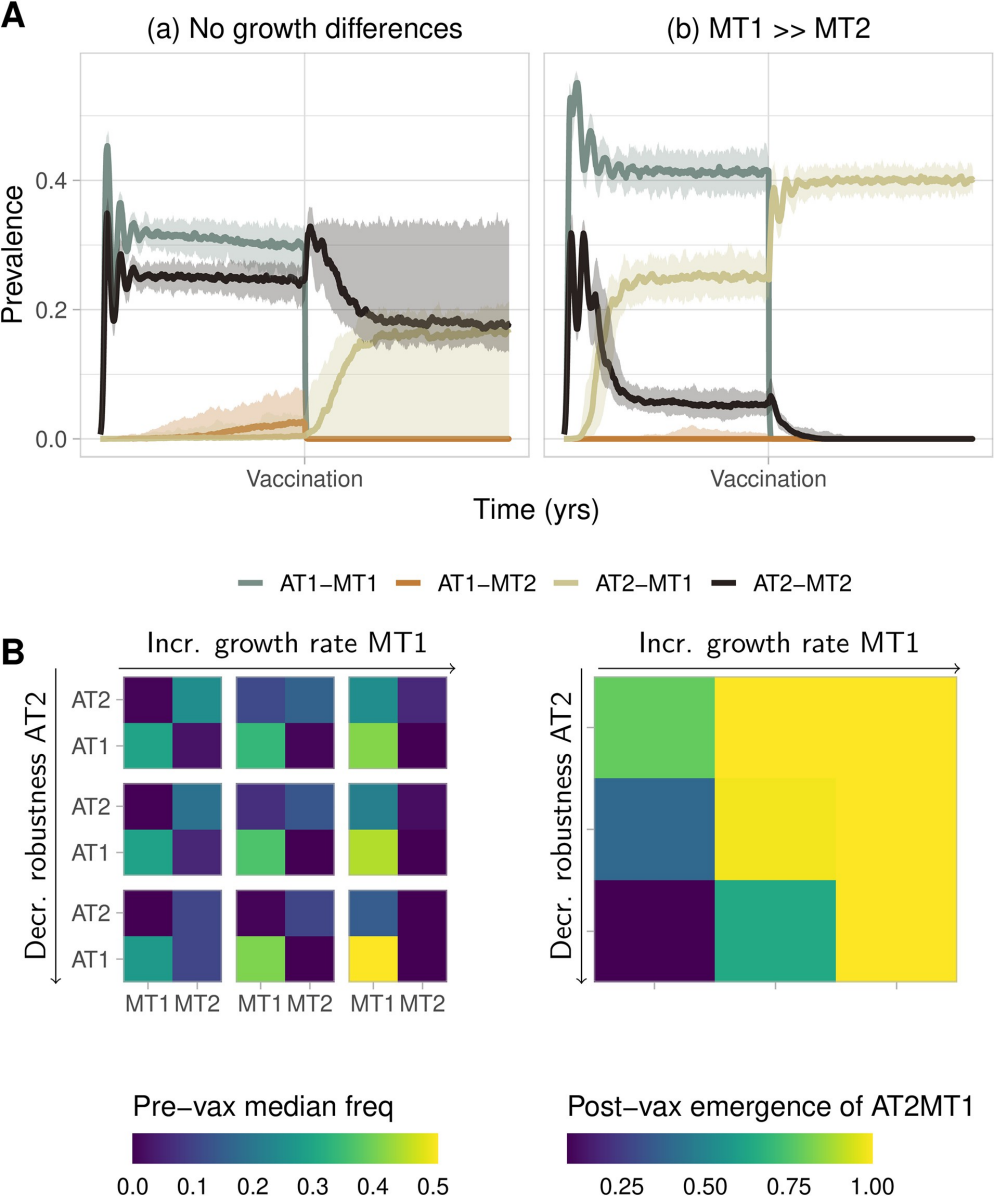
Strain structure is preserved for low growth differences among metabolic types. (A) Strain trajectories illustrating the pre- and post-vaccine dynamics when (a) there are no growth differences between MT1 and MT2; and (b) MT1 has a significant within-host advantage. Solid lines indicate median prevalence, shaded areas indicate 95% quantiles over 70 simulations. (B) Heatmaps summarizing trajectories over a range of parameters: *α*_1_ = 0.02, *α*_2_ ∈ [0.022, 0.025, 0.03], *κ*_2_ = 1.1, *κ*_1_ ∈ [1.1, 1.15, 1.2]. (Left) Median frequency of each AT/MT combination pre-vaccination. (Right) The fraction of simulations for which AT2-MT1 was present at the end of the simulation (post-vaccination). Simulations initialized with AT1-MT1 and AT2-MT2 only. Each simulation is run on 7000 hosts over 30 years, with vaccination at year 15. Additional parameters fixed: *τ* = 0.8, *p*_*τ*_ = 0.22. Full trajectories are shown in Fig A4 in S1 Text.

We next investigate the ability for differential fitness between metabolic and antigenic types to generate observed patterns of population structure over longer time-scales (Fig 4). In particular, we explore the conditions under which exclusion occurs between strains which share a metabolic type, and under which such a strain may emerge following the elimination of its competitor due to vaccination. We simulate two antigenic types AT1 and AT2, where AT2 is less fit than AT1, along with two possible metabolic types, MT1 and MT2. While we initialize the model with the strains AT1-MT1 and AT2-MT2, we allow “switches” to occur at a constant rate during a co-colonisation event. That is, whenever two distinct strains encounter each other within a single host, there is a fixed rate at which a new recombinant strain can emerge stochastically within that host. We vaccinate against AT1 after 15 years to observe the resulting vaccine-induced effects. That is, we examine when strains AT1-MT2 and AT2-MT1 are effectively excluded pre-vaccination, but where the recombinant AT2-MT1 has the possibility to emerge post-vaccination.

We observe that metabolic exclusion is predicted to occur as long as the within-host growth differential between metabolic types is small (Fig 4B). If neither metabolic type has a strong growth advantage, the initial strain structure (AT1-MT1 and AT2-MT2) is preserved, and recombinants (AT1-MT2 or AT2-MT1) are either unable to emerge or are strongly suppressed. This effect is diminished somewhat as the relative fitness among antigenic types is increased: as AT2 becomes weaker, there is decreased metabolic competition for the recombinant AT1-MT2, although its prevalence is suppressed through antigenic competition from the established strain, AT1-MT1. As the within-host fitness of MT1 increases, strain structure is eroded: eventually, the benefit incurred by acquiring the advantageous metabolic type overcomes the effect of metabolic competition, and all antigenic types become associated to MT1. However, if AT2 is extremely unfit, it may be unable to gain the beneficial metabolic type and strain structure is again preserved (provided the benefit from MT1 is not so large).

We furthermore investigate in which regimes the capsular switch variant AT2-MT1 emerges post-vaccination (Fig 4C). Here again, we find that the data is most consistent with only small differences among metabolic types, but potentially large differences among antigenic types. It is in this regime where we see strong patterns of metabolic exclusion pre-vaccination, yet the possibility for recombinants to emerge post-vaccination. The probability of such emergence depends strongly on the robustness of the non-vaccine AT. Conversely, in the regime where there are large differences in growth rates associated to different MTs, the effects of metabolic competition are mitigated, and we do not observe any vaccine-induced dynamics (other than the elimination of VTs).

We note that the parameters we vary here have distinct effects on both the strain’s within-host and between-host fitness. While small changes in the within-host growth rate lead to small changes in the population-level strain fitness in the *absence* of competition (Fig A5 in S1 Text), these same changes lead to significantly different behaviour in the systems studied here. In contrast, decreasing the robustness of an antigenic type will lead to a significantly decreased endemic equilibrium in a single-strain system, but we are able to observe metabolic exclusion over a range of values of this parameter.

**Fig 5:**
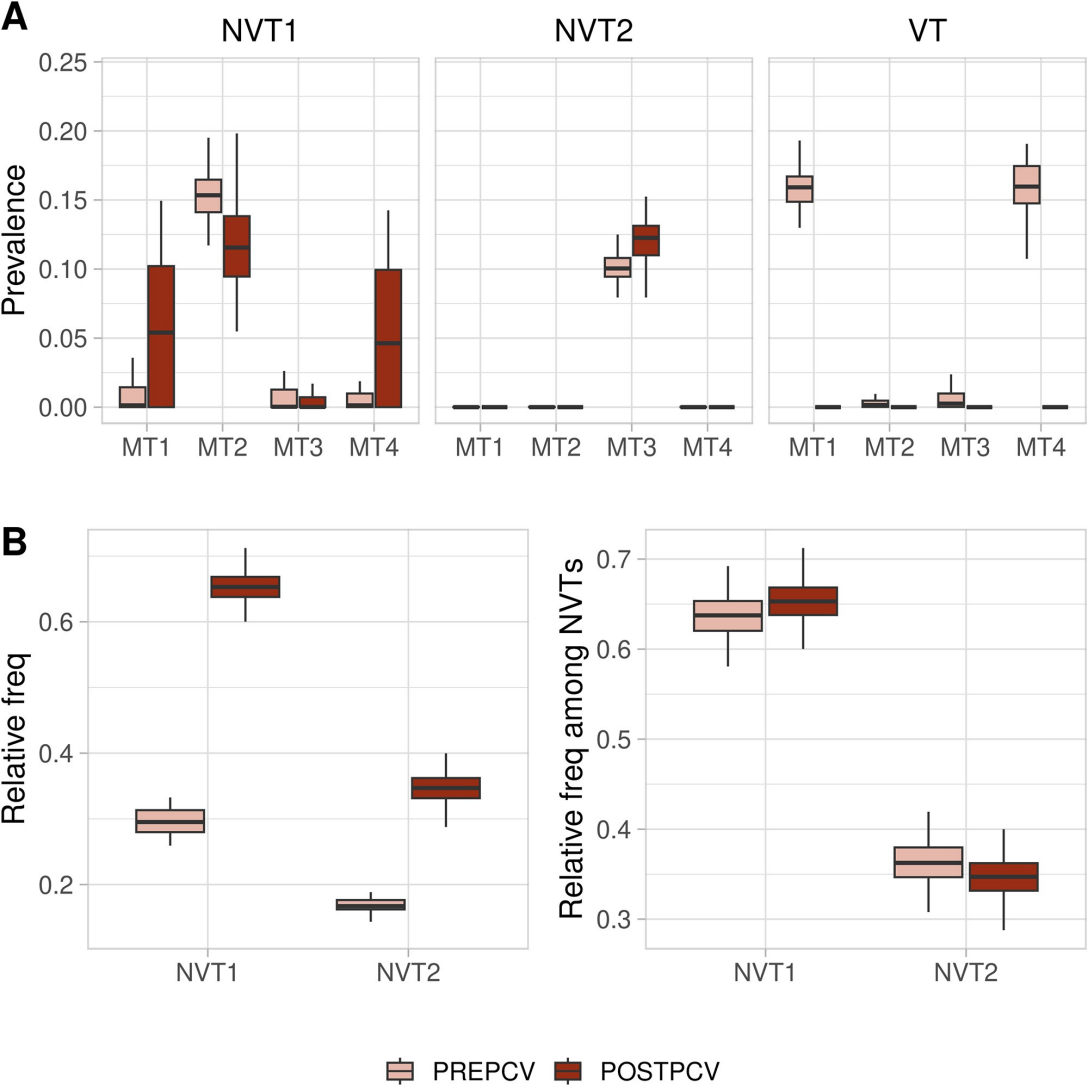
Pre- and post-vaccination dynamics with multiple NVTs. We fix the robustness of each antigenic type, with the VT being the most, and NVT2 being the least, robust. Simulations are performed on 7000 hosts over 30 years with vaccination at year 15. Each simulation is repeated 70 times. All strains are drug-susceptible. (A) Change in metabolic composition of each antigenic type following vaccination. (B) Change in relative frequency of both NVTs following vaccination (including the VT), compared to the change in relative frequency among NVTs only. Fixed parameters: *α*_*VT*_ = 0.02, *α*_*NVT*1_ = 0.025, *α*_*NVT*2_ = 0.03, *κ*_1_ = *κ*_2_ = *κ*_3_ = *κ*_4_ = 1.1, *p*_*τ*_ = 0.22, *τ* = 0.8, *β* = 0.09, *r*_*t*_ = 0.00005.

To illustrate these dynamics further, we investigate a three serotype system in Fig 5. We consider the case where NVT1 is much more robust than NVT2, but where all metabolic types have the same properties. Pre-vaccination, we see that metabolic exclusion occurs, with NVT1 being comprised predominately of MT2, NVT2 being predominately MT3, with the remaining metabolic types being associated to the VT. Following vaccination, we see that switching events may occur where NVT1 becomes associated to one or both of MT1 and MT4. Such events are not observed to occur within the less robust antigenic type. However, we show that while the metabolic composition of the NVTs may change, the overall post-vaccine prevalence for each NVT is roughly proportional to its pre-vaccination prevalence. That is, the relative frequency among NVTs does not change significantly following vaccination.

**Fig 6:**
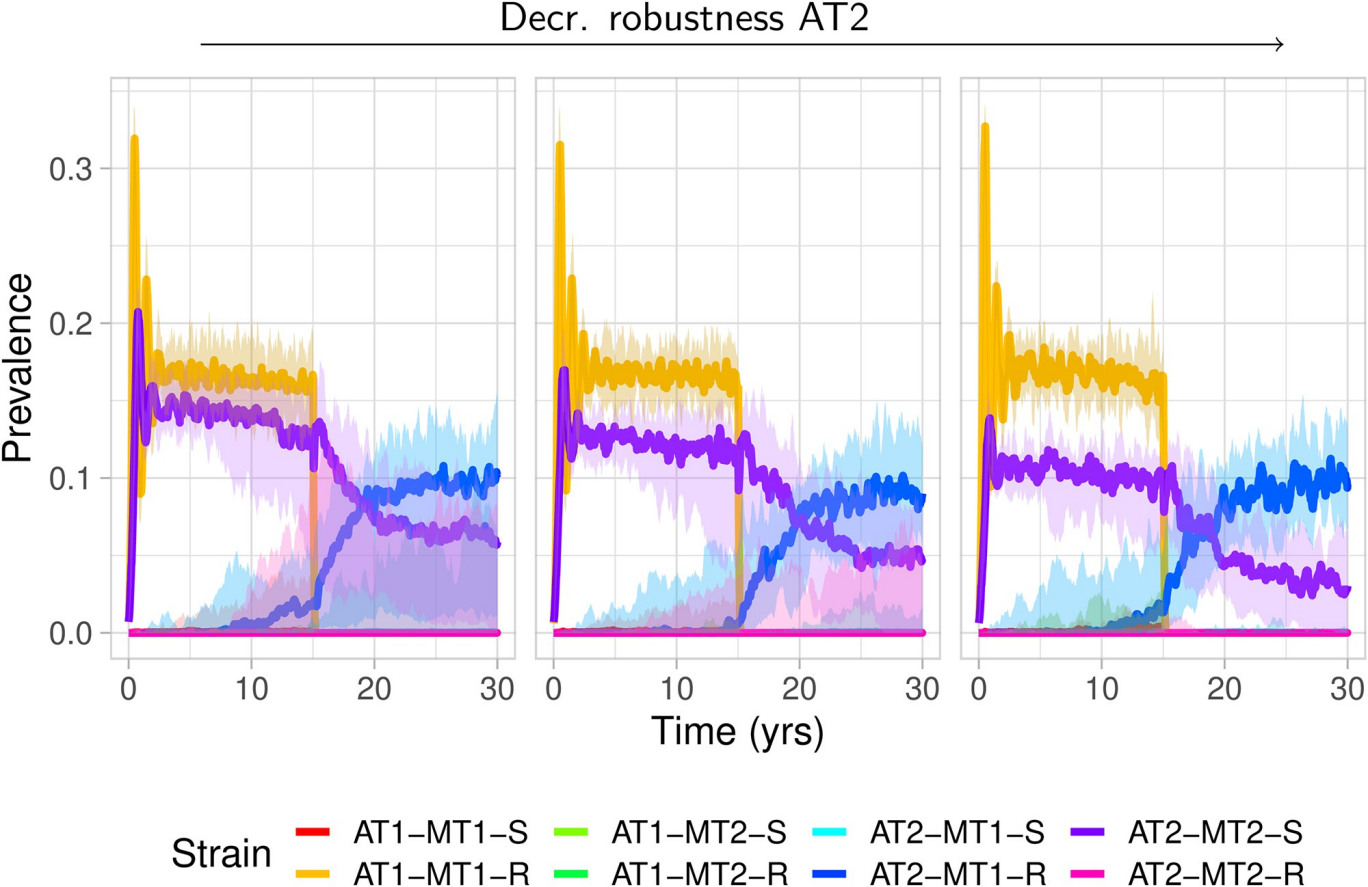
Post-vaccine success of resistant variant depends on antigenic properties. Each panel shows strain trajectories initialized with AT1-MT1-R and AT2-MT2-S, with vaccination against AT1 after 15 years. We only show here simulations in which MT1 was not eliminated following vaccination. The strength of immune response against AT2 is varied: *α*_2_ ∈ (0.026, 0.027, 0.028), with fixed *α*_1_ = 0.025. There is no difference in growth rates between MT1 and MT2. Resistance-specific parameters are fixed, with the growth cost of resistance *c* = 0.2, within-host treatment rate *τ* = 0.9 and population-level treatment coverage of *p*_*τ*_ = 0.34. Simulations are on 7000 hosts and repeated 72 times. Lines denotes median value with bands representing 95% quantiles.

We ask next how resistance may play a role in driving post-vaccine dynamics. In the absence of additional diversity, competition or fitness differences, we expect any emergent recombinant strain to reach, on average, equal frequency to that of its antigenic competitor. To investigate more complex patterns of post-vaccine replacement, we introduce a resistance type to each possible AT/MT combination. In addition to switching its antigenic type, we further allow a strain to gain a resistant locus in a co-colonisation event, or to lose the resistance locus at any time (see Methods for more detail). In Fig 6, we initialize each simulation with the strains AT1-MT1-R and AT2-MT2-S. Pre-vaccination, we see that a resistant variant is unable to establish itself within AT2. However, for those iterations in which MT1 was able to persist post-vaccination of AT1, the recombinant AT2-MT1-R is able to emerge. The ability for this strain to dominate within AT2 depends on the AT-dependent parameter *α*, which controls the host immune response. Here, we see a range of outcomes, with the relative frequency of the resistant (switch) variant generally increasing as the robustness of AT2 decreases.

We next investigate the effect of cross-immunity on our results. We are inspired once more by the pneumococcus, in which a number of serogroups have been defined on the basis of significant cross-reactivity [19]. While certain non-vaccine types (NVTs) within the same serogroup as a vaccine-targeted serotype were significantly affected by PCV7 (notably, serotype 6A), many NVTs within serogroups of vaccine types benefited significantly post-PCV7 and were associated with higher rates of capsular switching events (notably, serogroups 23 and 19 and serotype 6C) [7].

We implement a simple model of *natural* cross-immunity as a slight modification to the immune response in the within-host model (Eq 1). As before, we include all strains $A_{i}$ of the same antigenic type as strain *i*, and now we additionally look at strains within the same antigenic “group” as strain *i*, where the strength of cross-reactivity is modulated by the parameter *k*_*g*_ (see Eq 2). Note that here, naturally induced immunity is separate from vaccine-induced immunity, and this model does not predict the direct effect of vaccination within the antigenic group. Motivated by observations in the pneumococcus, we do not include any effect of vaccination within an antigenic group.

**Fig 7:**
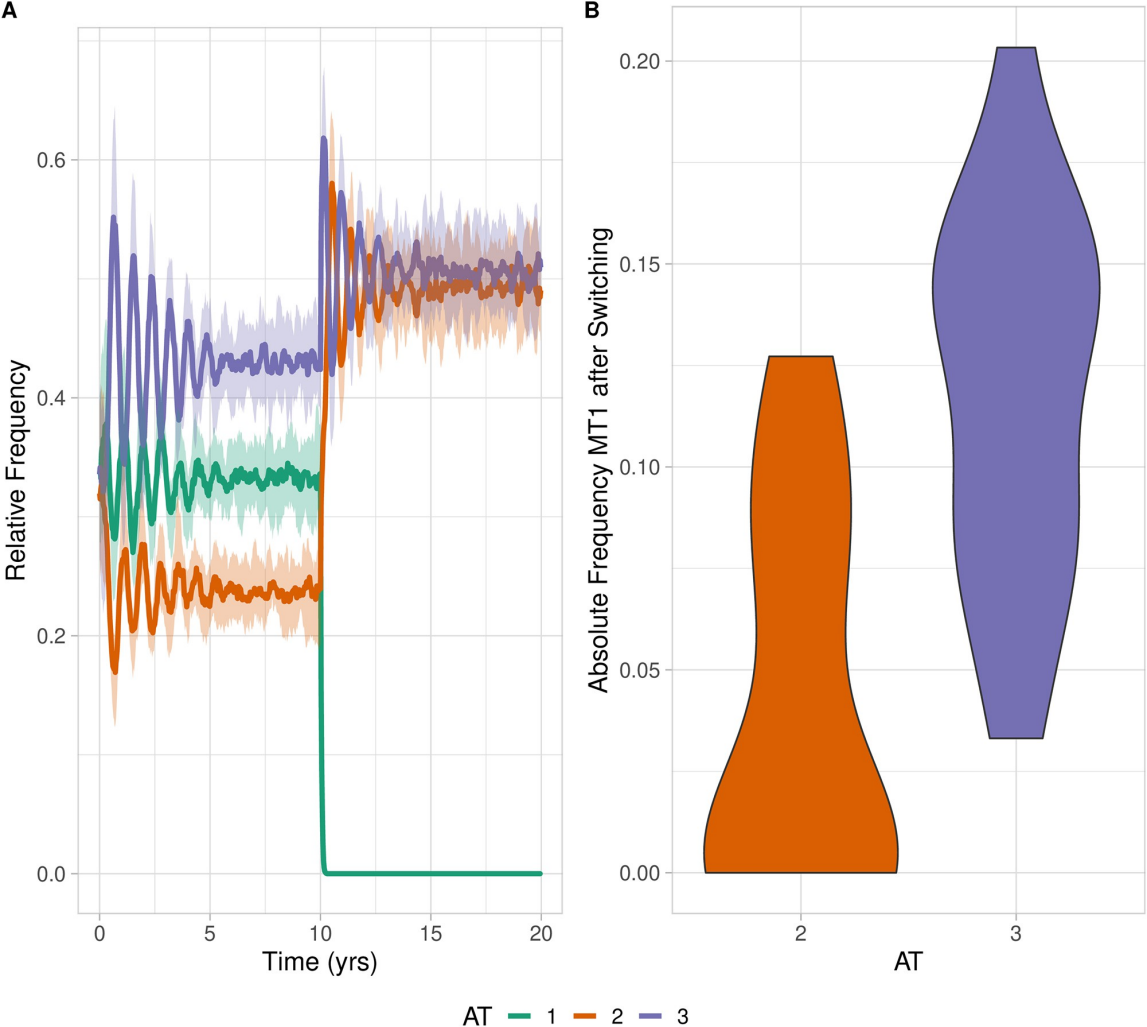
Three AT, three MT system with cross immunity between AT1 (vaccine type) and AT2 (non-vaccine type). Initialized with AT1-MT1, AT2-MT2 and AT3-MT3. MT1 is sometimes able to persist post-vaccination through switching events in either AT2 or AT3. (A) Relative frequency of each antigenic type (all strains of that type). (B) Absolute frequency of MT1 in each NVT (AT2 and AT3) provided a switching event occurred. Simulations on 10,000 hosts over 70 iterations. Fixed parameters: *k*_*g*_ = 0.1, *α*_1_ = 0.023, *α*_2_ = 0.024, *α*_3_ = 0.024, *κ*_1_ = *κ*_2_ = *κ*_3_ = 1.0, *p*_*τ*_ = 0.22, *τ* = 0.8, *β* = 0.09, *r*_*t*_ = 0.00005.

We illustrate the effect of cross-immunity in Fig 7. In this setup, there are 3 ATs and 3 MTs, with a total of 9 possible strains. There is (natural) cross-immunity between AT1 and AT2, with AT1 being a vaccine target and AT2 being a non-vaccine type, along with AT3. We initialize with AT1-MT1, AT2-MT2, and AT3-MT3, and vaccinate against AT1 after 10 years. As expected, the relative frequency of AT2 increases more significantly post-vaccination as compared to the unrelated type AT3. We seek to investigate whether or not this replacement coincides with an increased frequency of switching events. In Fig 7B, we see that MT1 is more likely to persist in the unrelated type AT3 than in AT2. Due to higher co-colonisation rates between AT3 and AT1 than AT2-AT1, MT1 is expected to first emerge within AT3, the non-related type. In the absence of additional, strong fitness differences between AT2 and AT3, the variant MT1-AT3 is often unable to emerge and succeed once MT1-AT2 has been established. This model, which has no preferential recombination within antigenic groups, therefore predicts that the frequency of switching events within antigenic groups would be lower than in unrelated antigenic types (in general).

## 3 Discussion

We demonstrate the importance of within-host competition on patterns of population structure and post-vaccine strain replacement in the pneumococcus. Data from pre- and post-vaccine populations exhibit competition and exclusion within putative metabolic types, and show how such types may be resilient to vaccination. Our modelling highlights how attributes linked to a strain’s antigenic and resistance profile are key drivers for post-vaccine strain success. Conversely, the model indicates that significant fitness differences between distinct lineages are less likely. This is because, as such differences become pronounced, the effect of metabolic competition is overshadowed and we do not observe the patterns of exclusion which are present in the data. Our results contribute to a mechanistic understanding of the work of Corander *et al*. [1], who highlight the importance of the accessory genome for predicting pneumococcal dynamics.

Our results expand upon those of Watkins *et al*. [5], who develop a compartmental model which exhibits metabolic exclusion and post-vaccine emergence of “switch” variants. This model which enforces strong within-host competition by either allowing strains to co-colonise without penalty or not at all. Here we investigate whether we can observe similar patterns of metabolic exclusion without this strong requirement. We instead model within-host competition explicitly through a dynamical model (Eq 1) and allow for co-colonisation and a complex trait space to affect within-host strain trajectories. We allow for a strain’s fitness to be affected by key within-host parameters, which in turn affect transmission. In this way, we allow for a strain’s within-host fitness, together with competition during co-colonisation, to feedback and affect its population-level fitness in an explicit way. This is in contrast to the model in [5], where a strain’s fitness can only be tuned through a direct transmission cost independently of within-host dynamics and competition.

While our model is inspired by direct observations of within-host dynamics [8, 9] and factors affecting duration of carriage [20], we primarily validate against population-level data over medium-to-long time-scales. Current deep-sequencing studies such as [8] are limited in their ability to identify within-host competition and take-over due, in part, to a low per-host sampling rate. However, advances in this area may allow for direct validation of models at the within-host scale in the future.

Our model predicts complex changes in associations between antigenic, metabolic and resistance profiles following vaccination. However, overall, each non-vaccine type is expected to maintain a relatively equal prevalence due to the strong effect of antigenic competition. Hence, a limitation of this framework is that we are unable to explain non-uniform patterns of serotype replacement in the pneumococcus [3]. Many instances of serotype replacement have been observed to occur within vaccine-related serotypes–that is, serotypes within the same serogroup as a vaccine type. This observation is perhaps surprising since it had been hoped that vaccine-induced immunity would provide partial protection against these types. A second interesting observation is that of the increased frequency of capsular switch variants arising within vaccine-related serotypes. A genetic basis for this observation has not been determined [7]. If we include natural (but not vaccine-induced) cross-immunity in our model, we indeed observe post-vaccine expansion of the vaccine-related type. However, this model does not predict an increase in the frequency of switching events between antigenically related strains. These results indicate that there may be a genetic basis for the increased frequency of switching events within serogroups in the pneumococcus.

Future work could explore this further by expanding the trait space of this model. We focus here on three particular traits, namely, a resistance-, metabolic- and antigenic-type. However, the flexibility of this model framework allows for the investigation of additional traits affecting competition and duration of carriage. This could include multiple forms of resistance (instead of the binary classification used here), overlapping metabolic profiles, or cross-immunity between antigenic types and an investigation into natural versus vaccine-induced immunity.

## 4 Materials and methods

The within-host model gives strain densities dependent on the competition between the co-colonising strains. This in turn affects the transmission process, which subsequently affects the host-level strain composition. This assumption, that competition and transmission take place on the same timescale and affect each other, necessitates the nested approach. This model does not have an implicit frequency-dependent selection, as we show in Fig A1 in S1 Text. All code necessary to reproduce our results is available at https://github.com/nmulberry/multistrainABM.

### 4.1 Within-host model

We develop a deterministic model to describe the dynamics of an arbitrary number of strains within a single host. This host-level model combines logistic growth with a simple model for a host immune response. Strains compete for resources if and only if they share the same metabolic type (MT). Thus, in contrast to the model of [5], we allow for strains of the same metabolic type to co-colonise, but competition modulates each strain’s ability to invade an established infection, and upon succeeding, the duration of carriage. Additionally, a strain’s antigenic type (AT) will affect the within host dynamics through previous host exposure to the AT, and through the strength of the immune response. We assume that resistance is associated with a growth cost [21], and allow for higher clearance of drug-sensitive strains if the host is on treatment.

Let *k* = 1, …, *N* denote the number of hosts and *i* = 1, …, *M* denote the number of strains. The set of strains with the same metabolic type as strain *i* is denoted by $M_{i}$, and similarly $A_{i}$ denotes the set of strains with the same antigenic type as strain *i*. The prevalence of strain *i* within host *k* at time *t* is $x_{i}^{k}\left(t\right)$, and $I_{i}^{k}\left(t\right)$ is the immune response to strain *i* in host *k* at time *t*. We define ${\vec{x}}^{k}$ as a fraction of the host’s carrying capacity, which we assume is equal among all hosts. The model equations are

$$\begin{array}{c} \begin{array}{ccc} \frac{d}{dt}x_{i}^{k}= & \kappa_{i}x_{i}^{k}(1-\sum_{j\in M_{i}}x_{j}^{k}) & \text{metabolic}\;\text{competition}\\ & -\alpha_{i}x_{i}^{k}I_{i}^{k} & \text{immune-mediated}\;\text{decay}\\ & -\tau_{i}\delta^{k}x_{i}^{k} & \text{treatment}\;\text{process}\\ & -\epsilon_{x}x_{i}^{k} & \text{natural}\;\text{decay}\\ \frac{d}{dt}I_{i}^{k}= & f(\sum_{j\in A_{i}}x_{j}^{k})-\epsilon_{I}I_{i}^{k}+g\left(I_{i}^{k}\right), & \text{basic}\;\text{immune}\;\text{response} \end{array} \end{array}$$ (1)

where

$$f\left(x\right)=\frac{x^{2}}{\rho^{2}+x^{2}}\;\text{and}\;g\left(x\right)=\frac{x^{2}}{\theta^{2}+x^{2}}.$$

The model for the AT-specific host immune response is based on Mayer *et al*. [22]. This simple, one-stage immune response is composed of an activation term *f* which depends on the density of the antigenic type within the host; a reinforcement term *g* which depends on the current strength of the immune response; and a decay term. All three terms are necessary in order to achieve a robust immune response which is able to eventually clear each strain from a host. In the case where we allow for cross-immunity, we modify the immune response to include strains within the same antigenic “group”, $G_{i}$ (modulated by parameter *k*_*g*_):

$$\frac{d}{dt}I_{i}^{k}=f(\sum_{j\in A_{i}}x_{j}^{k}+\sum_{j\in G_{i}}k_{g}x_{j}^{k})-\epsilon_{I}I_{i}^{k}+g\left(I_{i}^{k}\right).$$ (2)

As written in Eq 1, there are three strain-specific parameters: $\vec{\kappa }$, $\vec{\alpha }$ and $\vec{\tau }$. However, we make the following simplifying assumptions. First, the intrinsic growth rate, *κ*_*i*_, is determined by a strain’s resistance and metabolic profile. The treatment rate, *τ*_*i*_, of each strain is determined only by its resistance profile (with *τ*_*i*_ = 0 if strain *i* is drug resistant). The strength of the immune response against strain *i*, *α*_*i*_, depends only on its antigenic type. There is only one host-specific parameter, *δ*^*k*^, which indicates whether or not host *k* is on treatment.

The within-host model furthermore contains a number of additional parameters. The constant decay rates are fixed at *ϵ*_*x*_ = 0.03 and *ϵ*_*I*_ = 0.02. We fix *θ* = 70 and *ρ* = 0.01. Note that *ρ* corresponds to the cut-off strain density and will also be used in the transmission model described in the next section. These parameters are all fixed in order to achieve reasonable within-host dynamics and clearance on the desired time-scale of 1–4 months [20] (for a range of values *κ*_*i*_ and *α*_*i*_).

For a given strain, we compute the expected duration of carriage and immunity for a single infection in a single host. This is shown in Fig A2 in S1 Text as a function of *α*, and for fixed values of treatment rate *τ* and growth cost of resistance. For example, when colonisation is between 1–4 months, the corresponding duration of immunity (as defined by *I* > 0.001) is between 1.5–2 years. We ignore host-specific factors affecting duration of carriage, assuming that variation in carriage duration is primarily driven by pneumococcal genetic variation rather than host traits [20].

### 4.2 Transmission, transformation and treatment

At the population level, we model a homogeneous contact process (that is, we do not model any host-level contact heterogeneity) coupled with a treatment and recombination process.

Let *t*_*n*_ (*n* = 1, 2, …) be the start of each day in the simulation:

1. We assume that, for each strain, new infections occur as Poisson processes. For each strain *i*, we compute the number of transmissions of that strain as a Poisson random variable with rate $\beta \sum_{k}x_{i}^{k}\left(t_{n}\right)$.
2. For each transmission event, we choose a recipient host *k* uniformly at random, and set $x_{i}^{k}\left(t_{n}+\delta t\right)=x_{i}^{k}\left(t_{n}\right)+\rho .$ The parameter *ρ* is the minimum strain density.
3. Upon infection, the treatment status of the recipient host is updated with probability *p*_*τ*_.
4. Following transmission, we allow for transformation events to occur, modelled as Poisson point processes. At fixed rate *r*_*t*_ (where we typically take *r*_*t*_ = 0.00005), each of the following events may happen:
  - A strain can lose a resistance locus.
  - A sensitive strain may gain resistance from a resistant strain during co-colonisation.
  - A strain of one antigenic type can switch to become another antigenic type during co-colonisation.
5. Every *T* days, we sample one strain from each host, weighted by their relative within-host frequencies, to report.

We note that the recipients may already be infected or not, with the same or different strains, and may already have immune memory of the same antigenic type. We assume that the strain composition within a host does not directly alter the transmission dynamics, and that transmission depends only on the total density of that strain. An illustration of the model trajectories within a single host and at the population level over multiple iterations is shown in Fig A3 in S1 Text. Our results depend on the somewhat arbitrary rate *r*_*t*_; while this rate will quantitatively affect our results (primarily, the time-scale at which recombinants can emerge), our qualitative results are robust to reasonable values of this parameter.

### 4.3 Vaccination

At the time of vaccination, we reduce the growth rate of each vaccine type (VT) significantly, so that *κ* = 0.5 for each VT strain. Note that this is approximately half the typical value for *κ*. In this way, the vaccine type strains are eventually eliminated from the host population, but this process is gradual enough so as to allow for switching events to occur. Alternative approaches to modelling vaccination such as increasing host immunity were either less robust to strain elimination or eliminated the strains almost immediately. The goal of this model is to investigate competition among strains, and especially how elimination of vaccine types changes the system dynamics. We do not address questions such as when vaccination will be successful or not against a given type.

### 4.4 Data

**Fig 8:**
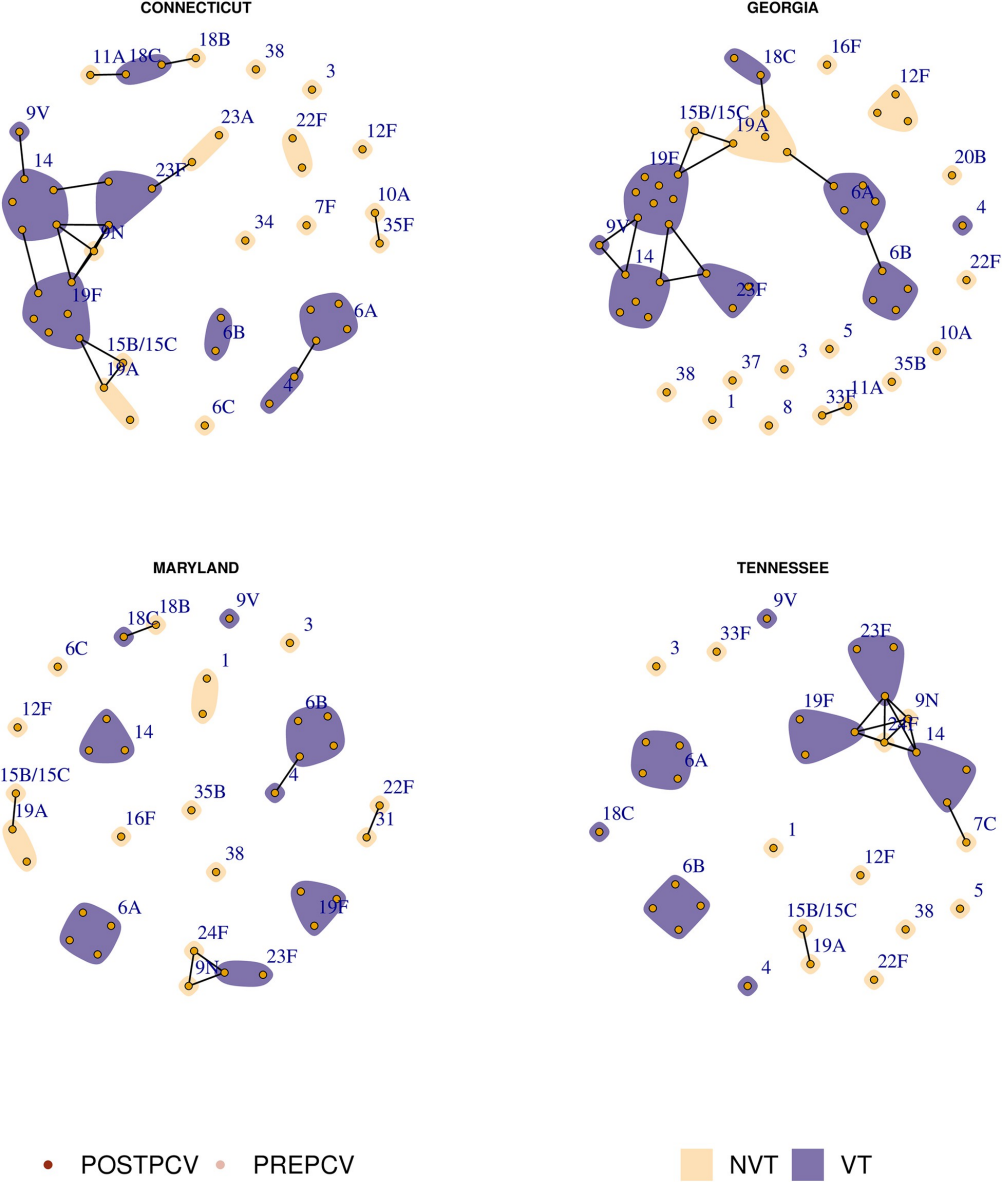
Pre-PCV7 sampled isolates across four US states. Each node denotes a distinct lineage (GPSC) within a serotype (labelled, and coloured according to PCV7 status). Edges indicate a shared lineage.

**Fig 9:**
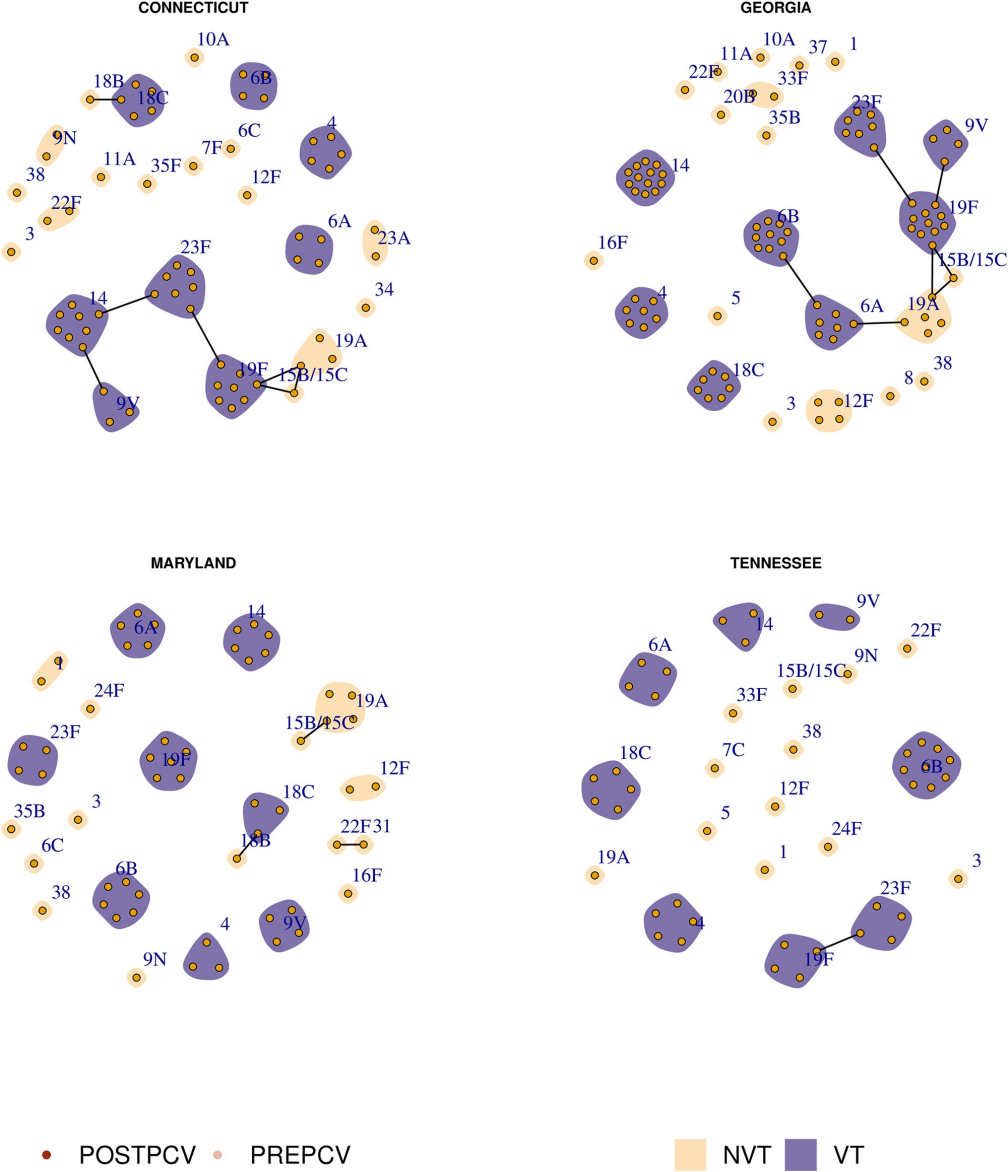
Pre-PCV7 sampled isolates across four US states. Each node denotes a distinct sequence type within a serotype (labelled, and coloured according to PCV7 status). Edges indicate a shared sequence type.

In our analysis of data collected from pneumococcal isolates as part of the Global Pneumococcal Sequencing project [23], we consider an isolate’s sequence type to be a proxy for a broader metabolic profile, following [5] and [18]. Global pneumococcal sequence clusters (GPSCs) are an alternate classification of isolates based on clustering performed on a core phylogeny of global sequences [16]. We might expect that these clusters, which indicate shared evolutionary history, may also be indicative of metabolic function and competition. In Fig 8, we show the pre-vaccination sampled population where each node is a GPSC and edges connect nodes with a shared lineage. As before, serotypes are denoted by communities which are coloured according to whether or not the serotype was targeted by PCV7. We compare these networks to those in Fig 9, which are identical except where nodes and edges are based instead on sequence typing. We see that stronger population structure emerges when we consider sequence types as opposed to the more broadly classified GPSCs.

## Supporting information

S1 Text Supplementary material. Additional analyses and results.
(PDF)
