# Supplementary material for: Pneumococcal population dynamics: Investigating vaccine-induced changes through multiscale modelling
Source: PLoS Comput Biol. 2023 Dec 28;19(12):e1011755. doi: 10.1371/journal.pcbi.1011755 (PMC10781023; doi:10.1371/journal.pcbi.1011755)
Supplement: S1 Text — Additional analyses and results. (PDF) [file pcbi.1011755.s001.pdf]

Pneumococcal population dynamics: investigating vaccine-induced changes through multiscale modelling

Supplementary Material

Nicola Mulberry, Alexander R. Rutherford, and Caroline Colijn

Department of Mathematics, Simon Fraser University, Burnaby, British Columbia, Canada

1 Model Neutrality

As discussed in Lipsitch et al. [1], co-infection models which have implicit within-host dynamics are at risk of violating ecological neutrality. Hence, a nested model with explicit within-host dynamics is well-suited to studying multistrain coexistence. It is readily recognized that the within-host model has the same behaviour whether or not a single strain is occupying a host, or if two identical strains (with the same antigenic and metabolic types) are occupying a host. Figure A1 shows these results at both the within-host and population-level scales. For both the within-host and nested model, the combined trajectory of two identical strains circulating is the same as that for just one of the strains. However, the criteria of Lipsitch et al. [1] require us to look also at the relative frequencies of each strain, with the condition that neutral null models should conserve the initial distribution of identical strains. Since this model is stochastic, the original strain frequencies are not necessarily preserved under identical strains. In a finite population and under no selection pressure, the strain frequencies are dominated by genetic drift. However, we can investigate this effect in the within-host model, to ensure that neither strain is given an inherent advantage. As shown in Figure A1, the within-host model preserves the initial frequencies of identical strains up until the minimum density cut-off is reached (at which point the absolute strain density would be very small).

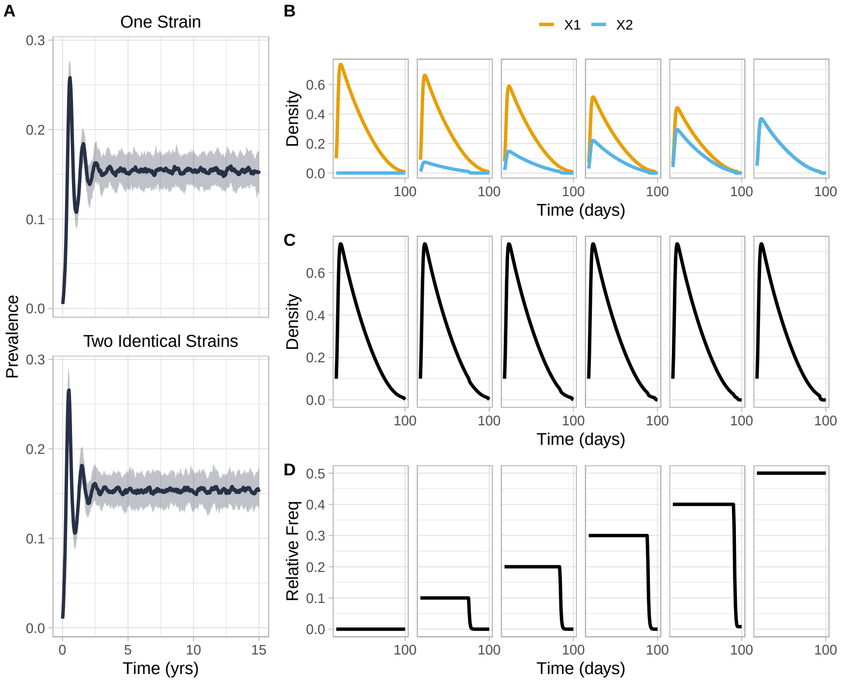

Figure A1: **The nested model exhibits neutrality**. (A) Population-level simulation Results with two circulating identical strains compared to a single strain. Simulations use 10000 hosts. (B) Within-host trajectories varying the relative frequency of two identical strains. (C) The total strain density within the host. (C) Relative frequency of strain 2, $X2/(X1 + X2)$, over the course of colonization.

2 Expected duration of carriage and immunity

We investigate the expected duration of carriage, and the resulting host immune response, in a single host under the deterministic within-host model. For $\alpha \in [0.02, 0.08]$, and for reasonable values of the resistance-dependent parameters c and τ, we achieve a realistic range for the expected duration of carriage [2, 3]. The resulting host immunity is robust enough to initially clear the strain, but also short enough to allow the strain to persist in the population and exhibit an endemic equilibrium.

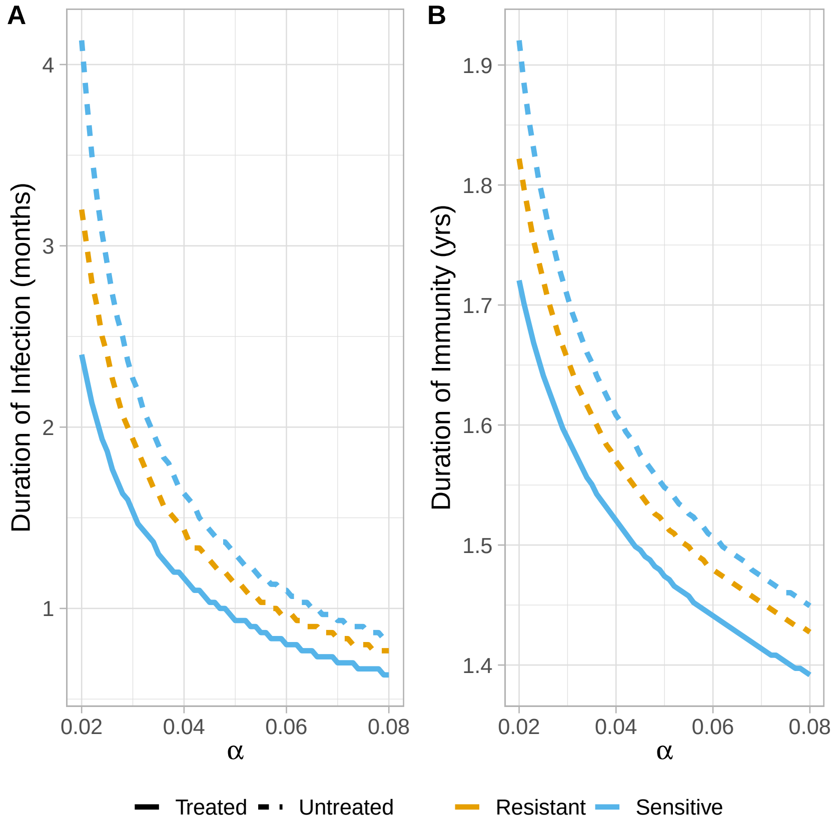

Figure A2: Expected duration of (A) carriage and (B) immunity in a naïve host. We consider a host to be infected (colonized) if the strain density $X > 0.01$, and we consider the host to be immune to reinfection of that strain if $I > 0.001$. Results are from running the within-host model with one strain and do not include co-colonization or re-colonization of the host. Fixed parameters: τ=0.35, $\kappa_S = 1.1$, $\kappa_R = 0.935$.

3 Within-Host Trajectories

Figure A3 shows the corresponding host trajectories from a single host in a single iteration of a full model simulation. Here, we model the two strains as fixed and do not allow for any gain or loss of resistance.

4 Additional trajectories

In Figure A4, we show the full trajectories for each run shown in the heatmaps in Figure 4 of the main text. As before, we simulate a system with two antigenic types (ATs) and two metabolic types (MTs) and vary (1) increasing the robustness of AT1 relative to AT2 and (2) increasing the within-host growth rate of MT1 relative to MT2.

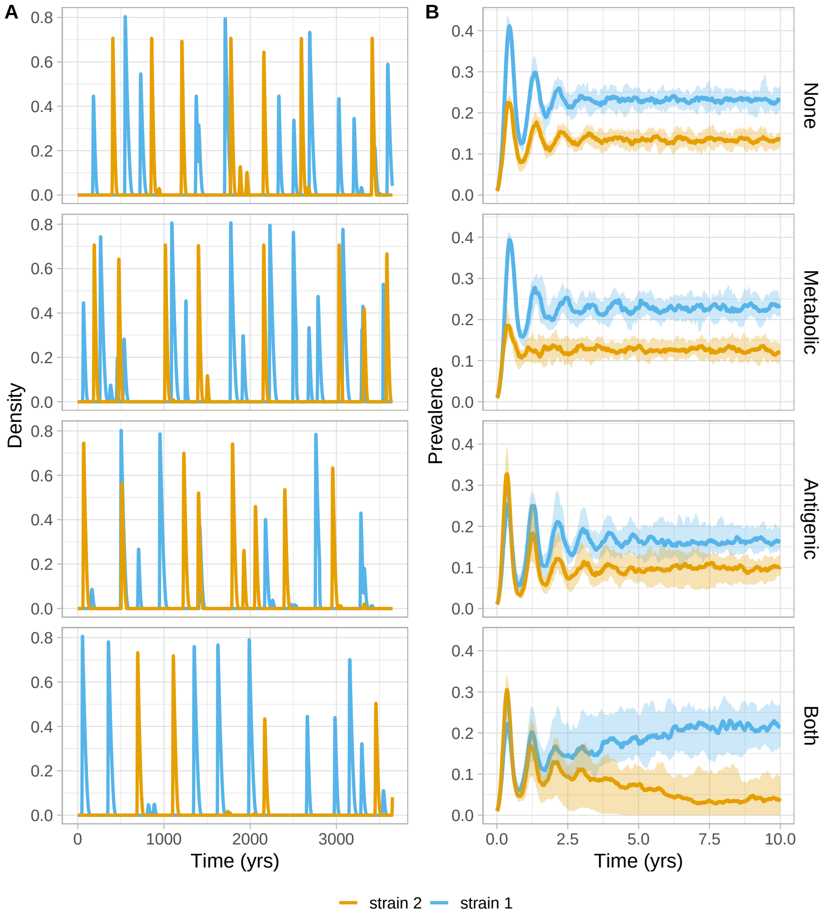

Figure A3: Comparison of host-level and population-level trajectories (no recombination). (A) Host-level trajectories. (B) The corresponding population level trajectories on 5000 hosts. Each simulation shows 2 strains, one sensitive and one resistant, with the corresponding competition type. Fix: c=0.15, $\tau = 0.35$, $p_\tau = 0.4$, $r_t = 0$.

We investigate the pre-vaccination trajectories further in Figure A5 by extending the pre-vaccination period to 50 years. In the leftmost column (with $\kappa_1 = 1.1$), we see a slight increase in the frequency of recombinant AT1-MT2 over this 50 year period relative to Figure A4, but we still do not see the pre-vaccine emergence of the NVT variant AT2-MT1. In the middle column (with $\kappa = 1.15$), in addition to the early rise of AT1-MT2, we also observe an increase in the frequency of the VT AT1-MT2 which is not seen after 15 years, and which coexists here with AT1-MT1. We would not expect this to affect the post-vaccine dynamics. The rightmost column (with $\kappa = 1.2$) is unchanged. Thus, while the pre-vaccination strain structure is not completely stable in all parameter settings, the main conclusions drawn with regards to strain structure after 15 years remain the same.

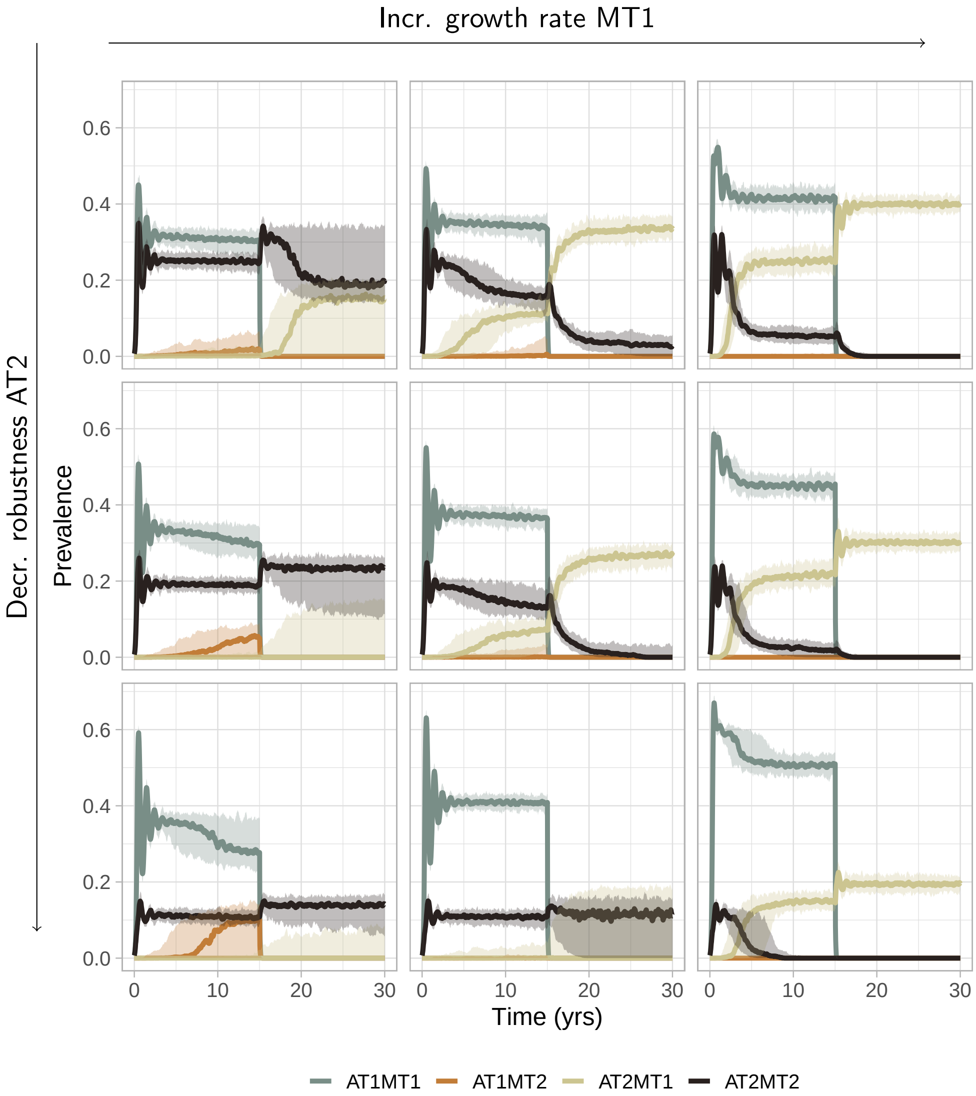

Figure A4: All trajectories corresponding to the heatmaps in Figure 4 of the main text. Solid lines indicate median prevalence, shaded areas indicate 95% quantiles over 70 simulations. Each simulation is run on 7000 hosts over 30 years, with vaccination at year 15. All simulations initialized with AT1-MT1 and AT2-MT2 only, with a fixed transformation rate. Vary the relative robustness of the antigenic types, $\alpha_2 \in (0.022, 0.025, 0.03)$, with fixed $\alpha_1 = 0.02$, and the relative within-host growth advantage of metabolic types, $\kappa_1 \in (1.1, 1.15, 1.2)$, with fixed $\kappa_2 = 1.1$. Additional fixed parameters: $\tau = 0.8, p_\tau = 0.22, \beta = 0.09, r_t = 0.00005$.

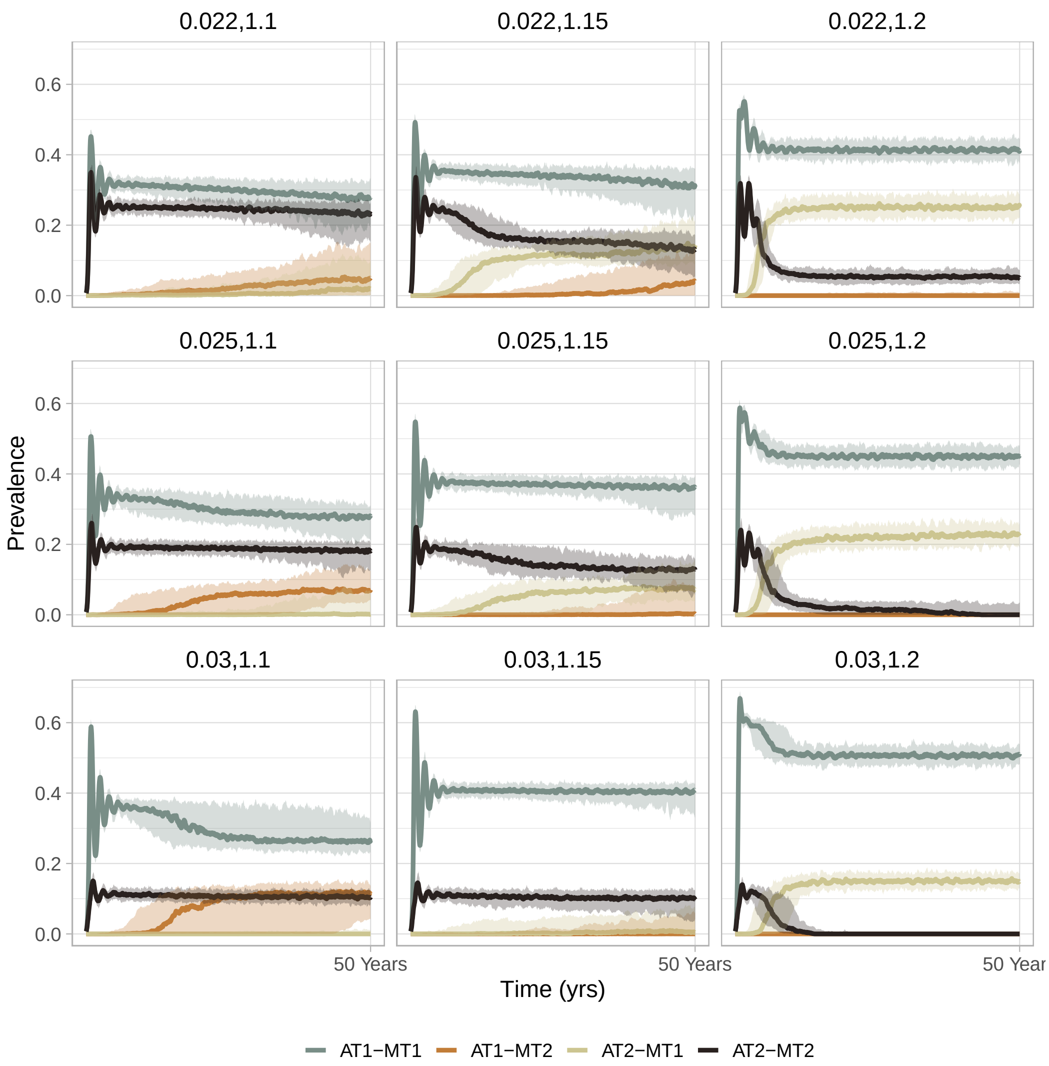

Figure A5: Trajectories corresponding Figure A4 with no vaccination and over a 50 year time-frame. Solid lines indicate median prevalence, shaded areas indicate 95% quantiles over 70 simulations. Each simulation is run on 7000 hosts over 50 years. All simulations initialized with AT1-MT1 and AT2-MT2 only, with a fixed transformation rate. We vary the relative robustness of the antigenic types, $\alpha_2 \in (0.022, 0.025, 0.03)$, with fixed $\alpha_1 = 0.02$, and the relative within-host growth advantage of metabolic types, $\kappa_1 \in (1.1, 1.15, 1.2)$, with fixed $\kappa_2 = 1.1$. The title of each tile indicates (α_2, κ_1). Additional fixed parameters: $\tau = 0.8, p_\tau = 0.22, \beta = 0.09, r_t = 0.00005$.

5 Fitness effect of within-host parameters

The results in Figure A4 depend on the AT-specific parameters α_i and the MT-specific parameters κ_i. To gain intuition on the expected fitness effect of each parameter under no competition, we show the median endemic equilibrium (i.e., at the host-population level) of a single strain for the range of (α, κ) values considered in the previous result. Thus, we see that in the absence of competition, increasing the robustness of the antigenic type for fixed κ increases the expected endemic equilibrium by up to 80%. In the results shown in Figure A4, the same jump in AT2 did not alter the behaviour of the system significantly in terms of generating observed patterns of population structure. However, an increase in κ from 1.1 to 1.15 for fixed α has a comparatively smaller effect on the between-host fitness of the single strain, but it results in a more significant change of the dynamics of the entire 4-strain system studied above.

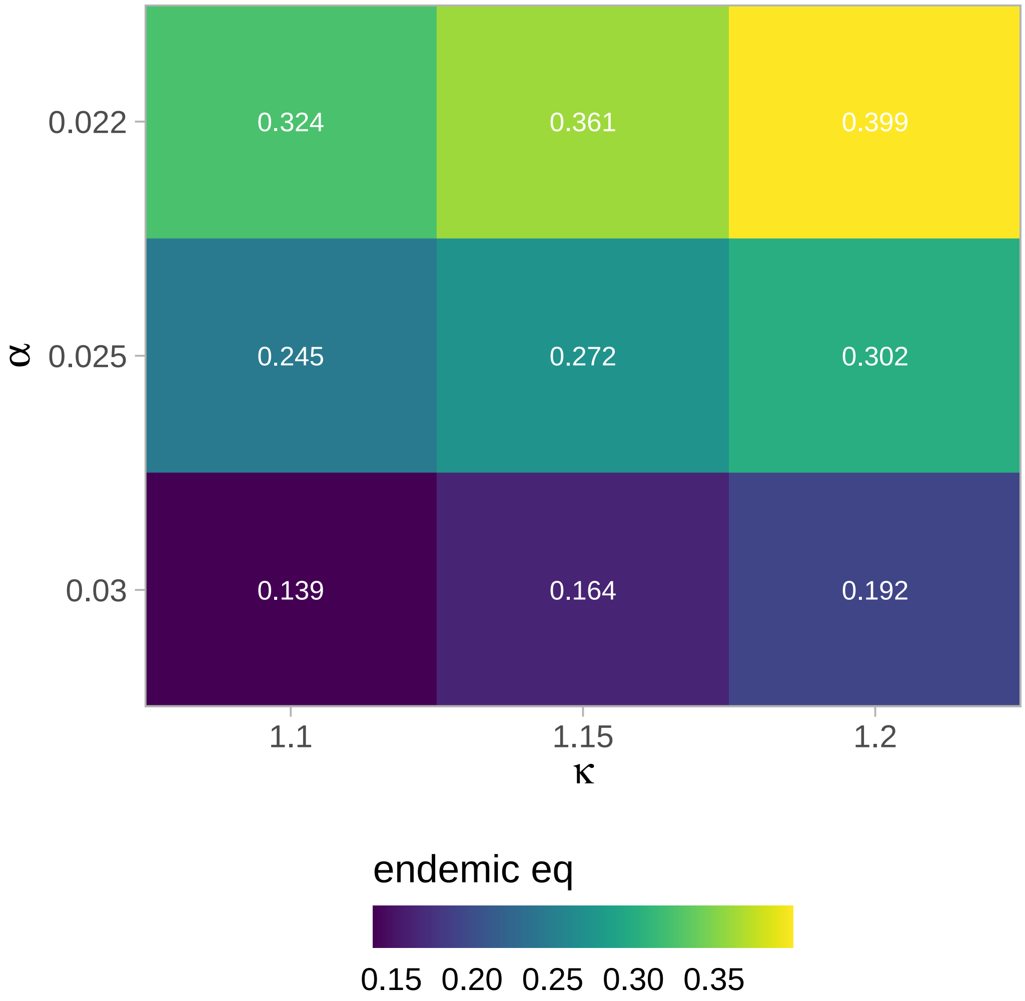

Figure A6: Effect of parameters α and κ on the single-strain (drug-sensitive) endemic equilibrium. Colour indicates median prevalence at 30 years over 70 simulations. Additional fixed parameters: $\tau = 0.8$, $p_\tau = 0.22$, $\beta = 0.09$, $r_t = 0$.

References

[1] Marc Lipsitch, Caroline Colijn, Ted Cohen, William P Hanage, and Christophe Fraser. No coexistence for free: neutral null models for multistrain pathogens. *Epidemics*, 1(1):2–13, 2009.

[2] John A Lees, Nicholas J Croucher, David Goldblatt, François Nosten, Julian Parkhill, Claudia Turner, Paul Turner, and Stephen D Bentley. Genome-wide identification of lineage and locus specific variation associated with pneumococcal carriage duration. *Elife*, 6:e26255, 2017.

[3] Karen L Sleeman, David Griffiths, Fiona Shackley, Linda Diggle, Sunetra Gupta, Martin C Maiden, E Richard Moxon, Derrick W Crook, and Timothy EA Peto. Capsular serotype–specific attack rates and duration of carriage of streptococcus pneumoniae in a population of children. *The Journal of Infectious Diseases*, 194 (5):682–688, 2006.
